# Understanding Barriers to Effective Injury Care by Medical Trainees and Traffic Law Enforcement First Responders in Low-Income Contexts in Uganda (Motor Registry Project Part 2): Convergent Mixed Methods Analysis

**DOI:** 10.2196/84774

**Published:** 2026-06-10

**Authors:** Herman Lule, Benson Oguttu, Micheal Mugerwa, Michael Lowery Wilson, Jussi P Posti

**Affiliations:** 1 Injury Epidemiology and Prevention (IEP) Research Group, Turku Brain Injury Centre, Division of Clinical Neurosciences, Turku University Hospital and University of Turku, Vähä Hämeenkatu 1B, Turun Yliopisto, Turku, FI20500, Finland, 358 465699821, 358 23132737; 2 Center for Health Equity in Surgery and Anesthesia (CHESA), Institute of Global Health Sciences, University of California San Francisco, San Francisco, CA, United States; 3 Department of Surgery, Kiryandongo Regional Referral Hospital, Kigumba, Uganda; 4 Department of Public Health, Botswana-Havard Health Partnership, Gaborone, Botswana; 5 Department of Public Health Sciences, California Baptist University, Riverside, CA, United States; 6 Section for Oral Health, Heidelberg Institute of Global Health (HIGH), Heidelberg University, Heidelberg, Germany; 7 Neurocenter, Department of Neurosurgery and Turku Brain Injury Center, Turku University Hospital, Turku, Finland; 8 Division of Clinical Neurosciences, Neurosurgery, University of Turku, Turku, Finland

**Keywords:** emergency medical services, health care disparities, medical training, wounds and injuries, mixed methods

## Abstract

**Background:**

Trauma-related mortality exhibits a marked social gradient, driven by access barriers and health inequities worldwide. These barriers jeopardize progress toward Sustainable Development Goals 3 and 4.

**Objective:**

This study aimed to investigate the prehospital and in-hospital barriers to timely injury care as perceived by frontline trainee physicians and traffic law enforcement professionals during real-time treatment execution in Uganda. Additionally, we aimed to highlight the potential impact of these barriers on trauma outcomes.

**Methods:**

This study used a convergent mixed methods approach. Qualitative data were collected through structured interviews and face-to-face focus groups with diverse teams of 500 frontline trainee physicians, including surgical residents, interns, medical students, and traffic law enforcement professionals. Directed content analyses for structured interviews and focus groups were conducted in NVivo (version 14, release 2023; QSR International). Quantitative data were concurrently collected using a survey questionnaire from the Motorcycle Trauma Outcome Registry, which included 1003 patients with trauma. We categorized barriers as prehospital or in-hospital barriers and as trauma team-related, patient-related, or health system–related barriers. Multilevel restricted maximum likelihood logistic regression analyses of factors associated with barriers to injury care were analyzed using Stata (version 15.0; StataCorp LLC). Odds ratios (ORs) and 95% CIs were reported; statistical significance was defined as *P*<.05.

**Results:**

Qualitative analyses identified key prehospital barriers, including delays in emergency medical services activation, ambulance arrival, and transportation. In-hospital barriers were primarily shortage of supplies, delays in identifying life-threatening injuries, and insufficient critical care services. Quantitatively, among the 1003 audited patients with trauma, 42% (416/1003) faced barriers during treatment. The most common obstacles were delays in treatment decisions (232/1003, 23%) and securing necessary supplies (180/1003, 18%). The presence of barriers was independently associated with a 3-fold increased likelihood of unfavorable Glasgow Outcome Scale scores (OR 3.15, 95% CI 2.23‐4.66; *P*<.001) for neurological injuries and was linked to a 4-fold increase in odds of 90-day mortality (OR 4.20, 95% CI 2.25‐6.94; *P*<.001). After adjusting for injury severity and clustering effects by hospital teams and resources, the presence of barriers was associated with arrival by public means (adjusted OR [aOR] 1.62, 95% CI 1.09‐2.41; *P*=.02), increasing age (aOR 1.01, 95% CI 1.00‐1.03; *P*=.01), sustaining 1 or more injuries requiring admission (aOR 1.92, 95% CI 1.18‐3.14; *P*=.01 vs aOR 3.69, 95% CI 1.95‐6.98; *P*<.001), and a severe Kampala Trauma Score of ≤6 (aOR 2.71, 95% CI 1.37‐5.37; *P*=.004).

**Conclusions:**

Multiple barriers to trauma care are more frequent for severe injuries and are associated with poorer neurological outcomes and higher mortality. These findings indicate the need for targeted, multifaceted interventions that incorporate frontline health workers’ perspectives to improve trauma care delivery in low-resource settings facing both prehospital and in-hospital barriers.

## Introduction

Globally, injury-related mortality is characterized by significant socioeconomic gradients [1]. These social inequalities manifest through suboptimal prehospital care access, fragmented health care systems, and suboptimal postcrash care, including the unmet need for physical and mental rehabilitation after injuries, in both low- and high-income countries (HICs) [2]. For instance, research highlights substantial regional disparities in trauma-related mortality rates, particularly in Europe, where Eastern Europe experiences rates nearly 3 times higher than those in Western Europe [1,3]. In the United States, compelling evidence demonstrates desperate trauma outcomes that are linked to disparities in ethnicity and insurance coverage, where uninsured persons of low socioeconomic status are the most vulnerable to adverse injury outcomes, whereas in Australia, rural geographic disparities to injury care are well documented [4]. Research from Canada mandates that issues of health care inequities be addressed by medical trainees and well-established physicians through their civic duties as patients’ advocates [5].

In Asia and Africa, the burden of injury is disproportionately higher in nations with poor economies, contributing to approximately 4.5 million premature deaths annually in low- and middle-income countries (LMICs) [3,6,7]. For instance, it is estimated that 36% of injury-related deaths in LMICs are linked to delays or barriers to injury care [8]. These barriers are highlighted in the United Nations General Assembly resolution 74/299, with a rapidly approaching target deadline: to halve road traffic crash–related global mortality and ensure well-being for all individuals by 2030, congruent to Sustainable Development Goals (SDG3) [9]. However, whereas recent trends show declining inequalities in injury care access among HICs, in LMICs, socioeconomic inequities, inadequate infrastructure, and shortage of human resources for health have continued to exacerbate these challenges, resulting in disparities in access to injury care both between and within regions, for instance, in urban versus rural areas [3,6,10].

In Africa, the disparities in injury care are manifested by suboptimal emergency medical services coverage in most countries in the region [11,12]. For instance, despite the high volume of trauma cases in Uganda and Rwanda, significant challenges persist, including inadequate insurance coverage and limited resources for trauma care. These challenges also encompass a weak prehospital care system characterized by shortage of ground ambulances and evacuation of injured patients predominantly by traffic police, a scarcity of trauma specialists, and the constraints of rigid, nondecentralized referral systems [13,14]. These issues not only impact injury outcomes but also have consequences on the learning and clinical experiences of medical students and resident doctors who train in such environments, including burnouts and poor retention of the health care workforce, after graduation from medical schools [15]. Medical training and capacity building have been identified as top priorities for addressing African health system challenges [15]; however, research that positions trainee physicians as policy advocates for improving the social determinants of health at individual and practice levels, particularly through identifying barriers that impact the care of patients in their regular work environment, is limited.

Positioning medical trainees as patient advocates is essential for building a competent health workforce, particularly in LMICs where resource constraints worsen health disparities and create imbalances in geographic and specialty distributions. Current academic programs in Uganda focus primarily on biomedical education, failing to connect medical training with community-based interprofessional practice, social determinants of health, and population needs. These connections are crucial for preparing future physicians to assume progressive responsibilities aligned with community priorities. Systematic reviews indicate that integrating experiential learning, reflective practice, and feedback in education fosters long-term professional identity formation, empowering trainees to influence local and national health policies, which is vital for addressing the dual burden of disease and workforce deficits in LMICs [16,17].

We previously reported on a Motorcycle Trauma Outcome Registry (MOTOR) project that considered medical trainees and traffic law enforcement first responders as sustainable human resources for injury care in rural settings with a positive impact on injury outcomes through trauma team training [18,19]. This ancillary study formed an integral component of tertiary outcomes to supplement the MOTOR project by detailing the perspectives of these cadres regarding barriers to injury care in low-resourced contexts.

Coping with patient care demands, sufficient health system resources, and an enabling work environment are essential for the success of medical trainees and frontline health workers in their learning and patient care responsibilities. These demands intensify when caring for patients with trauma due to the emergency and life-threatening nature of their injuries. Therefore, it is crucial to understand the barriers that may hinder effective treatment of such patients from the perspectives of trainee physicians, who serve as frontline health workers, and from traffic law enforcement professionals who work in the capacity of paramedics in low-resource settings. However, existing studies often lack a comprehensive analysis of the barriers to injury care faced at the individual patient level, which are influenced by geographical and cultural contexts [13,14,20]. In Uganda, studies that have attempted to investigate some of these barriers have limited their scope to prehospital factors mainly in urban settings and have excluded insights from frontline health care workers such as trainee physicians who predominate the health system workforce [21,22]. This phenomenon can be attributed, in part, to the challenges associated with acquiring information from key frontline stakeholders. These challenges arise from their demanding work schedules, remote geographical locations, privacy concerns, and the aspiration to manage the heterogeneity of research participants, which often proves to be realistically unattainable.

Therefore, the aim of this study was to identify the prehospital and in-hospital barriers to timely injury care as perceived by trainee physicians and traffic law enforcement professionals who are the first point of contact and are actively engaged in the care of injured patients in a low-resource context, as well as to elucidate how these barriers impact injury outcomes. We structured the identified barriers into 2 frameworks, focusing on both prehospital and in-hospital challenges, while examining both infrastructural and human resource factors. Additionally, we aimed to delineate the specific barriers encountered at both the individual-patient and trauma-team levels during the real-time execution of treatment.

## Methods

### Study Design

This was a convergent mixed methods study nested within a broader MOTOR project, which investigated the impact of rural trauma teams and training on outcomes of motorcycle-related injuries [18]. To effectively address the complex interconnected geopolitical, infrastructural, and human resources issues that delay the execution of lifesaving interventions, mixed methods research was essential to fully understand the specific barriers to timely injury care in low-resource settings, particularly from the perspectives of health care personnel involved in the initial patient contact. Prior research had identified mixed methods as particularly suitable for investigating complex health care systems [23]. In their recent systematic review, Whitaker and colleagues [20] have recommended that future research assessing barriers to injury care should use innovative mixed methods to investigate both prehospital and in-hospital delays, as well as the associated care processes and outcomes. In this study, we fitted a 2-framework approach into the concept of 3 temporal delays in accessing treatment within the context of LMICs, including seeking, reaching, and receiving injury care, as described by Vaca et al [24]. This theoretical framework permitted the convergence of qualitative and quantitative data to gain a deeper understanding of the obstacles to injury care and the characteristics of patients who experience these barriers. The research was documented in accordance with good reporting of mixed methods studies guidelines [25], whereas the qualitative element was reported in line with the COREQ (Consolidated Criteria for Reporting Qualitative Research) [26].

### Study Settings

The study was conducted from September 2, 2019, to August 27, 2023, at 6 regional tertiary hospitals in Uganda: Jinja, Hoima, Fort Portal, Mubende, Kiryandongo, and Kampala International University Teaching Hospital, which also serve as surgical residency and internship training centers. Multidisciplinary teams of consultant surgeons, residents, interns, and medical students operate the trauma and casualty units. Patients with trauma typically arrive via ground ambulances, traffic police patrols, or public transport, where they are assessed and treated by the multidisciplinary teams. These facilities operate as level III trauma centers and collectively serve a population of 18 million across 50 districts and 3 major cities in Uganda.

### Study Population and Source of Data

Qualitative data were collected from surgical residents, interns (doctors or nurses), and third- and fifth-year medical students associated with the surgery departments of the participating hospitals. Additionally, we engaged law enforcement professionals specializing in road traffic regulations, who serve as first responders to road traffic–related injuries within the municipalities that constituted the catchment areas of these hospitals. The qualitative data were collected through investigator-administered structured interviews and audio-recorded face-to-face focus groups. The structured interviews had coded predetermined themes regarding barriers to injury care identified from previous literature and featured open-ended options to cater for emerging new themes [8,20]. These interviews were administered as part of a pretest conducted prior to the development training of rural trauma teams previously described for the frontline health workers [27,28]. On the other hand, quantitative data were gathered from investigator-administered survey questionnaires for patients who sustained motorcycle-related injuries, received care at any of the participating hospitals during the study period, and participated in the MOTOR project [18,29].

### Sample Size Estimation and Sampling Methods

Given the complexity of the research topic and the cultural, geographical, and professional diversity of our study population, we aimed to recruit 50-80 participants for structured interviews from each of the 6 geographically distinct hospitals. We consecutively recruited samples of surgery residents, intern physicians, nurses, medical students, and purposively selected traffic rescue police involved in immediate contact with injured individuals. Participants were contacted from those who had opted into the rural trauma team development training project to complete the 15-minute structured interviews [18]. Participants who completed the structured interviews were subsequently recruited for 40-minute focus groups, organized into 100 teams. Each focus group comprised 5 participants and was managed by 3 research team members: HL administered the interviews, MM recorded field notes, and BO moderated the group sessions, verbalizing new responses verbatim. The groups consisted of varying compositions: rescue police alone (N=20), medical trainees alone (N=20), or a mix of both (N=60). Each participant was recruited only once. This data triangulation approach aimed to further explore the context of emerging themes through follow-up questions and to stimulate new themes based on team dynamics, aligning with an initial strategy to incorporate these groups into rural trauma teams [28].

To minimize institutional and selection bias, a transparent eligibility criterion was established. Trainee participants were stratified by hospital resources capacity and by study year as a rough estimate of expertise, followed by random assignment into 5-member groups using a blinded lottery method. Additionally, bias was further mitigated by triangulating data from focus groups and interviews and by continuously assessing data saturation to ensure a diversity of experiences.

We assumed data saturation and ceased recruitment when no new information, themes, or insights emerged in the monitoring log after 3 consecutive interviews and across different groups at various study sites, in accordance with previous studies [30,31]. Theme saturation was achieved after conducting 15-17 focus groups at each of the 6 hospitals, totaling 100 groups with 434 medical professionals and 66 traffic law enforcement officers (total sample=500). Data collection was capped upon reaching saturation to optimize time and resources, thereby avoiding redundancy while ensuring the comprehensiveness of emerging themes from diverse professional contexts [32]. Systematic reviews indicate that depending on the complexity of the research question and diversity of the research population, 9-17 interviews are sufficient for qualitative studies involving homogeneous populations, while a minimum of 50 interviews may be appropriate for culturally diverse populations [30,31].

The quantitative component assessed barriers to injury care for 1003 patients with trauma treated at the 6 study sites. The quantitative sample size was derived from an open-source R Shiny application for the MOTOR trial, which was a cluster randomized, parallel controlled trial with discrete time decay correlation structures for multiple periods as described by Lule et al [18].

### Eligibility Criteria

The qualitative component included trauma care frontliners, such as chief surgical residents, medical interns, traffic police officers stationed on major highways for immediate road traffic accident responses, and medical students in surgical and traumatology clinical rotations. Medical participants without prior exposure to surgery or emergency department rotations were excluded, as they were presumed to lack experiences with patients with trauma and the associated barriers to care. Additionally, participants who anticipated being unable to complete both the structured interviews and focus groups due to commitments, such as police officers on duty, were also excluded to ensure comprehensive capture of each participant’s experiences regarding the studied phenomenon.

The quantitative component included patients aged 2-80 years who had sustained neurological or musculoskeletal injuries resulting from a motorcycle crash and attended the emergency departments of the 6 study sites within 24 hours during the study period. To minimize confounding from injury mechanisms and comorbidities affecting neurocognitive and musculoskeletal outcomes, we excluded participants with a documented history of stroke and neuropsychiatric disorders, as outlined in the study protocols [18,27].

### Data Collection

Data were collected and precoded by global surgery and doctoral researchers HL and MM according to themes identified in the existing literature [20]. The data collection process was conducted simultaneously using pretested tools [18,28,29]. The qualitative component was part of a broader face-to-face patient-public consultative engagement for the MOTOR project [18]. After explaining the research objective and the procedure for completing the structured interviews, we asked participants to identify, assess, and rank the barriers. Subsequently, we used focus groups to gain an in-depth understanding through follow-up questions, to probe novel barrier themes, and to fully capture the experiences of participants (Multimedia Appendix 1). This information was intended to inform the establishment of emergency medical services, trauma teams, and trauma registries focused on motorcycle crash–related neurological and musculoskeletal injuries, which represent Uganda’s most significant trauma burden [33].

In the quantitative component, a prospective MOTOR trial project was established at 6 regional referral hospitals in Uganda [18,19], which captured quantitative data as detailed in Multimedia Appendix 2. Consultants, surgical residents, and interns involved in trauma care documented barriers encountered during the execution of definitive injury care for 1003 patients with trauma (MOTOR participants). Data on demographics, injury severity based on the Glasgow Coma Scale (GCS), and the Kampala Trauma Score (KTS) [34] were collected at baseline. On the other hand, trauma outcomes, including all-cause mortality, the Glasgow Outcome Scale (GOS) for traumatic brain injuries [35], and the Trauma Outcome Measure Scale (TOMS) score [36] for musculoskeletal injuries, were recorded 90 days postinjury.

Quantitative data were captured using precoded paper-based questionnaires and later uploaded to the secure REDCap (Research Electronic Data Capture; Vanderbilt University) electronic system hosted by Turku University in Finland [37]. Information was captured if a barrier was encountered during the execution of treatment, and each documented barrier triggered an alert, prompting an audit of the patient’s case file during weekly virtual meetings via Zoom (Zoom Communications, Inc). These 15- to 30-minute meetings, summarized in field notes, clarified the nature of the barriers and included verbatim anonymous documentation of responses from research assistants (designated 2-year experienced medical officers) and attending clinicians. Corrections were made for any inaccurate entries identified during the audits. This comprehensive approach aimed to capture a nuanced understanding of barriers affecting injury care access in real time to enhance future interventions.

### Data Analysis

We analyzed data from each component of this mixed methods study independently. The basis of this approach was to enrich the interpretation of the main outcomes of the MOTOR trial project in a broader context.

### Qualitative Analyses

Directed content analysis for both structured interviews and focus groups was conducted in NVivo (version 14, release 2023; QSR International). We used an iterative approach to track and document the prevalence of emerging themes using a log of nodes in NVivo. The results from primary qualitative data for both structured interviews and focus groups were consistently noncontradictory; thus, the overall triangulated results from these 2 qualitative approaches were presented. Where applicable, verbatim responses were presented as quotes to provide further context from open-ended questions.

### Quantitative Analyses

Concerning prehospital barriers (delay 1), participants selected from 6 responses. Provided with scores ranging from a minimum of 1 to a maximum of 6, participants ranked the barriers using a 6-point Likert scale, where 1 indicated “not a barrier,” 2 “very weak barrier,” 3 “weak barrier,” 4 “averagely weak barrier,” 5 “strong barrier,” and 6 “extraordinarily strong barrier.” A barrier category was considered significant (major theme) if it attained an overall mean score exceeding the prespecified hypothesized average of 3.5; otherwise, it was classified as a minor theme.

For in-hospital team barriers (delay 2) and system barriers (delay 3), there were 4 potential responses presented to medical participants, with Likert scores ranging from 1 (“least important barrier”) to 4 (“very important barrier”). While initial qualitative data collection targeted emergent themes, Likert items were incorporated with open-ended questions in a mixed methods design to enrich qualitative inquiry and facilitate triangulation with quantitative data. Predetermined Likert response categories (eg, “not a barrier” to “very important barrier”) quantified complex qualitative constructs such as attitudes, perceptions, and preferences. These response patterns informed purposive sampling for follow-up qualitative interviews across demographic groups and clinical experience levels. This approach bridged quantitative precision and qualitative depth by elucidating reasons behind each of the numeric Likert choices. The barrier category was considered important (major theme) if it surpassed the prespecified hypothesized average of 2.5.

The overall ranking of barriers was determined by calculating weighted mean scores, derived from the sum of individual Likert component scores multiplied by the number of responses for each component, subsequently divided by the valid sample size. This methodology has been previously validated for assessing barriers to trauma care in LMICs [20]. For example, in relation to prehospital delays, responses were scored on a Likert scale as follows: 1 (not a barrier), 2 (very weak barrier), 3 (weak barrier), 4 (averagely weak barrier), 5 (strong barrier), and 6 (extraordinarily strong barrier).


$$\begin{array}{rl} \text{Weighted mean barrier score} & =\left[1(n_{1})+2(n_{2})\;+3(n_{3})+4(n_{4})+5(n_{5})+6(n_{6})\right]\\ ÷N \end{array}$$

where *n*_1_=number of people responding per component, and *N*=sample size.

To analyze data from the MOTOR, first, we dichotomized the responses into 2 categories, that is, whether a barrier was present (yes or no). Second, whenever a barrier was identified, responses were further categorized into three categories: (1) team-related barriers that lead to delays in the identification, prioritization, or timely referral of life-threatening injuries by the emergency teams; (2) individual barriers, either from patients or from clinicians, that contributed to delays in decisions regarding surgery, initiation of treatment, or referral of injuries exceeding local capacity; and (3) in-hospital system barriers that caused delays in securing essential supplies, including oxygen, anesthetics, sutures, and blood products, or delayed access to a functional laboratory, imaging diagnostics, theater space, or intensive critical care services. These categories were subsequently represented as proportions based on their relative contribution to the total ranks. The mean rank scores for each barrier across various cadres (third- and fifth-year medical students, intern, and police) were compared with the hypothesized mean using the nonparametric Kruskal-Wallis equality of proportions rank test.

Finally, to determine factors associated with treatment barriers and their impact on injury outcomes, we assessed normality with the Levene test and performed bivariate and multilevel restricted maximum likelihood mixed-effects logistic regression analyses using Stata (version 15.0; StataCorp LLC) after adjusting for clustering effects. The mvaghermite integration method was used to explore potential correlations between barriers and injury outcomes, including all-cause mortality and morbidity at 90 days postinjury, as measured by the GOS [35] and TOMS [36]. Confounding and effect modification were evaluated using Cochran-Mantel-Haenszel statistics. Variables demonstrating a *P* value of <.2 in the Mantel-Haenszel strata and those with biological relevance to injury severity were included in the final model.

Recalling that the severity of injury is an important determinant of its outcome, the effects of barriers on injury outcomes were stratified by injury severity at baseline based on the GCS and KTS II for neurological and musculoskeletal injuries, respectively [34]. The selected outcome tools were chosen due to the primary focus of the MOTOR trial project on neurological and musculoskeletal trauma [18], which represent the most significant injuries resulting from motorcycle crashes [33]. For neurological injuries, outcomes were dichotomized as favorable (if GOS score 4‐5) or unfavorable (if GOS score 1‐3), whereas for musculoskeletal injuries, outcomes were dichotomized as favorable (if TOMS equaled or exceeded initial Trauma Expectation Factor Score) or unfavorable (if TOMS was less than Trauma Expectation Factor Score), congruent to previous studies [35,36]. A detailed description of how these tools were used to evaluate the morbidity associated with injuries, including assessments for pain, physical function, disability, satisfaction with treatment, and overall quality of life after injury, is reported in the MOTOR trial protocol [18].

### Convergence

Qualitative responses were analyzed in conjunction with quantitative metrics for each construct by 2 independent researchers (HL and MM). Any contradictions identified during this analysis were addressed by a third researcher (BO). The convergence status was categorized as “convergent” if the qualitative findings aligned with the quantitative results, “complementary” if the combined outcomes were assessed as synergistic, and “divergent” if the 2 results were inconsistent. Divergence indicated contradiction, as reflected in the notation (Qualitative≠Quantitative). In contrast, complementarity examined the different aspects of a phenomenon from multiple perspectives, leveraging the strengths of each method to cover more content than either method alone, thereby providing fuller, richer, multidimensional, yet nonoverlapping insight (Quantitative+Qualitative). Conversely, the convergent approach required consistency of outcomes across methods (Qualitative=Quantitative).

### Ethical Considerations

For this research involving human participants, all methods were performed in accordance with the ethical standards stipulated in the declaration of Helsinki and its later amendments, and in accordance with the relevant local guidelines and regulations. Ethical approval was obtained from the Research and Ethics Committees of Mbarara University of Science and Technology (reference: 1/7; 05/5‐9) and the Uganda National Council for Science and Technology (reference: SS 5082). All participants or their legally authorized representatives provided written informed consent prior to enrollment into the study. We ensured confidentiality and anonymity of participants through the use of codes and generic pseudonyms, such as “doctor,” “nurse,” or “police” in the reports. Each participant was compensated with transport reimbursement of US $7.50 as approved by the research and ethics committee.

## Results

### Qualitative Findings

Of the 500 trauma care frontliners who participated in structured interviews and focus groups for qualitative analysis (100% response rate), 27% (135/500) were females, 73% (365/500) were males, 13% (66/500) were traffic law enforcement officers, and 87% (434/500) were medical trainees.

### Prehospital Barriers (Delay 1)

**Figure 1.**
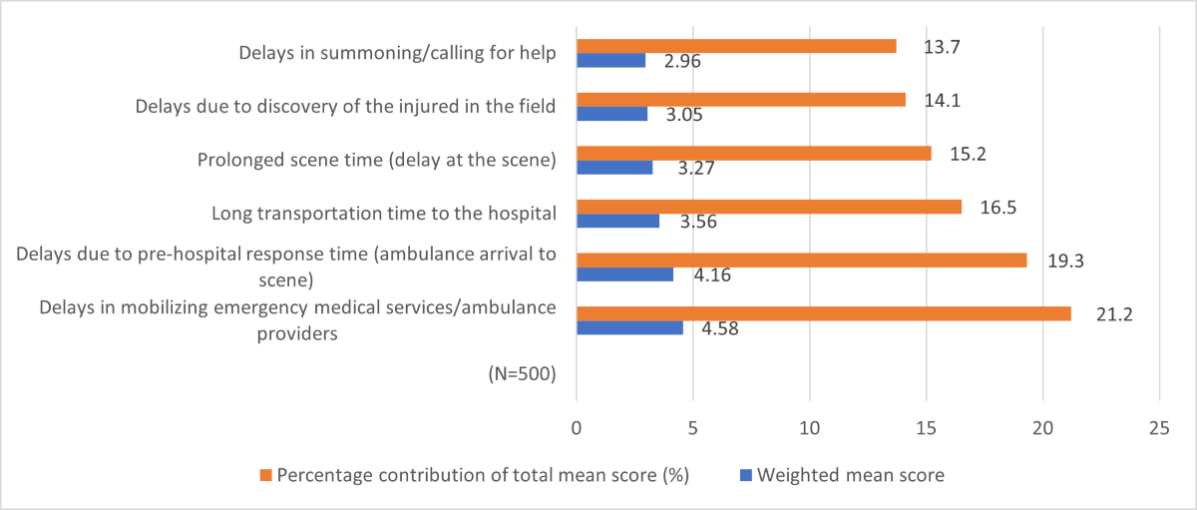
Overall weighted mean scores for prehospital barriers (delay 1) ranked from lowest to highest.

The 3 highest ranked barriers to accessing injury care identified in the assessment of prehospital factors were as follows: delays in the activation and mobilization of emergency medical services (105/500, 21%); delays attributable to prehospital response times, including the arrival of ambulances at the scene (95/500, 19%); and prolonged transportation times to the hospital (75/500, 15%) (Figure 1).

We found that while the police evacuated the patients with trauma, there were no designated rural networks of emergency rescue police to intervene for traffic crash victims.

*The role of evacuation of traffic crash victims is not specifically designated to particular officers within our jurisdiction which means any road traffic officer on duty can oversee such cases*.

[Participant no. 2, traffic law enforcement officials]

In addition, limited human resource capacity was reported as a key obstacle for the law enforcement professionals to participate in prehospital evacuation of injured patients.

*Often we are understaffed and not able to undertake transfer responsibilities timely as this would mean leaving one’s station vacant which would not be acceptable in our line of duty*.

[Participant no. 36, law enforcement official]

Furthermore, a lack of key focal people concerned with coordinating emergencies at recipient hospitals and suboptimal communication technologies was cited as a key barrier to timely injury care.

*Of course, you want to communicate timely but the poor communication technologies and network breakdown will let you down. Our intercom is not linked to that of the hospital emergency departments unless one is chanced to have physical contact there. Nevertheless, we are required to drop them (patients) by the government emergency hospital departments*.

[Participant no. 11, traffic police commander]

We established that there were also concerns of bias and mistrust that led to nondisclosure of injury-related information, which could delay the emergency evacuation process and insurance compensation claims.

*We would be delighted to have an integration of emergency evacuation rescue services that is well coordinated along with the health ministry, I guess this would increase our public trust. You want to help evacuate these accident victims, but they think you are interrogating, arresting, and prosecuting them for their traffic crimes*.

[Participant no. 47, police officer]

### In-Hospital Trauma Team and Individual-Patient Barriers (Delay 2)

**Figure 2.**
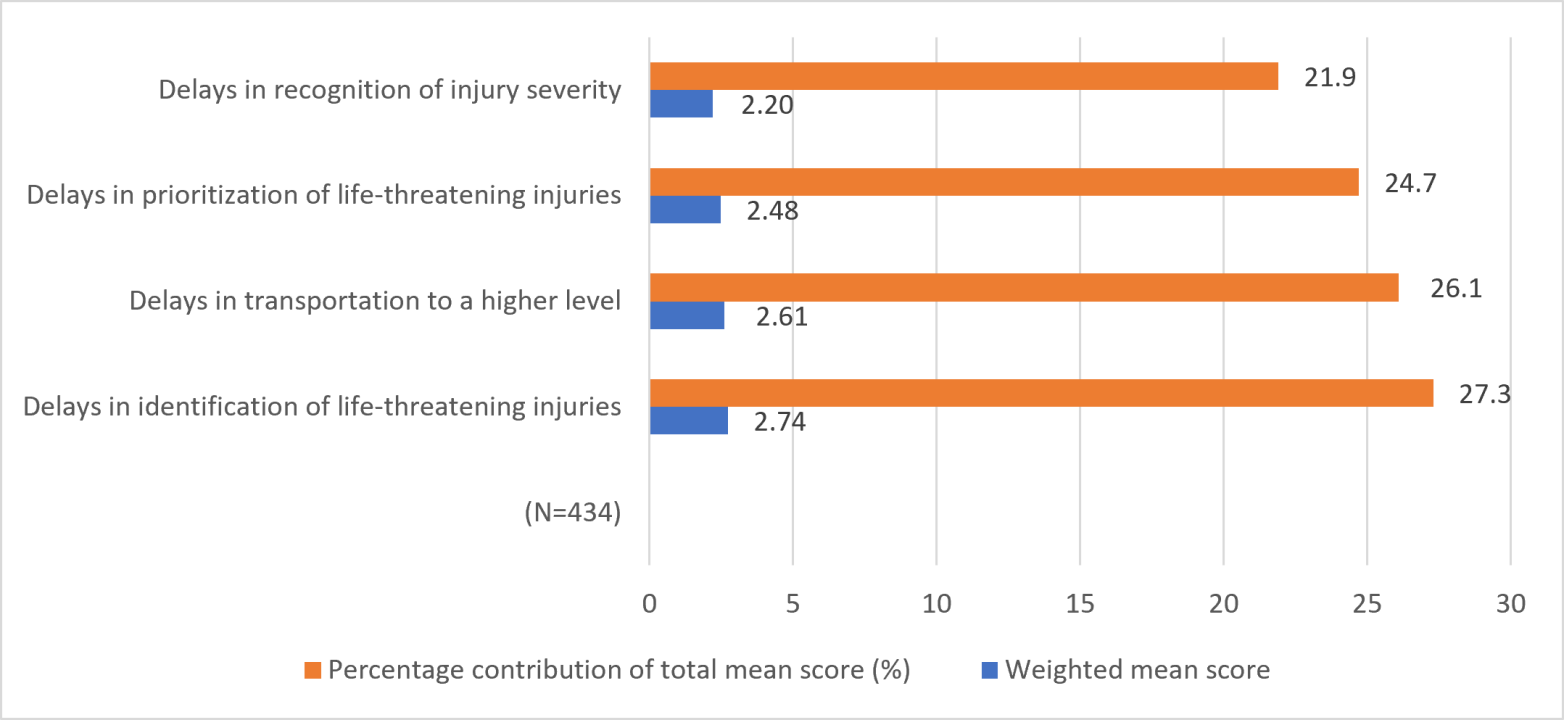
Overall mean weighted scores for in-hospital trauma team barriers (delay 2).

The highest ranked in-hospital team barriers were delays in identification of life-threatening injuries (117/434, 27%) and in executing referrals to higher trauma centers (113/434, 26%) (Figure 2).

We found that significant financial limitations prevented patients from obtaining emergency laboratory or imaging investigations that required out-of-pocket payments at private facilities, contributing to unnecessary delays. Additionally, there were no clear referral protocols linking the public to private facilities for specialized investigations or interventions that were not available in public facilities.

*We wanted to obtain head and brain CT scans, but when the service in our government national referral hospital was down, the patient’s next of kin delayed mobilizing financial resources to outsource one from private. We do not have direct referral pathways to private hospitals so then we had to wait until patients’ relatives availed us with the results of the investigations before operating*.

[Expert participant no. 1, attending neurosurgeon]

The trauma teams from qualitative clinical audits raised concerns about delays in hospital arrivals due to reliance on public transportation and dysfunctional ambulance systems. Additionally, there was limited capacity among emergency medical services staff and critical care supplies for life-threatening injuries, compounded by suboptimal health insurance coverage and communication barriers with the families of the injured individuals.

*He arrived late in a taxi as there was no fuel in the ambulance from the referring hospital. The staff there say were understaffed and were unable to accompany the patient as no one would cover their duty. They could have placed a chest tube though, but they were out of stock. Our ICU is full though, so we plan to refer him to a private hospital after a chest tube, but we are facing difficulties with direct communication, he has no attendants available yet as his family is still mobilizing resources for ICU costs. He does not seem to be insured*.

[Expert participant no. 3, attending anesthesiologist]

### In-Hospital System Barriers (Delay 3)

**Figure 3.**
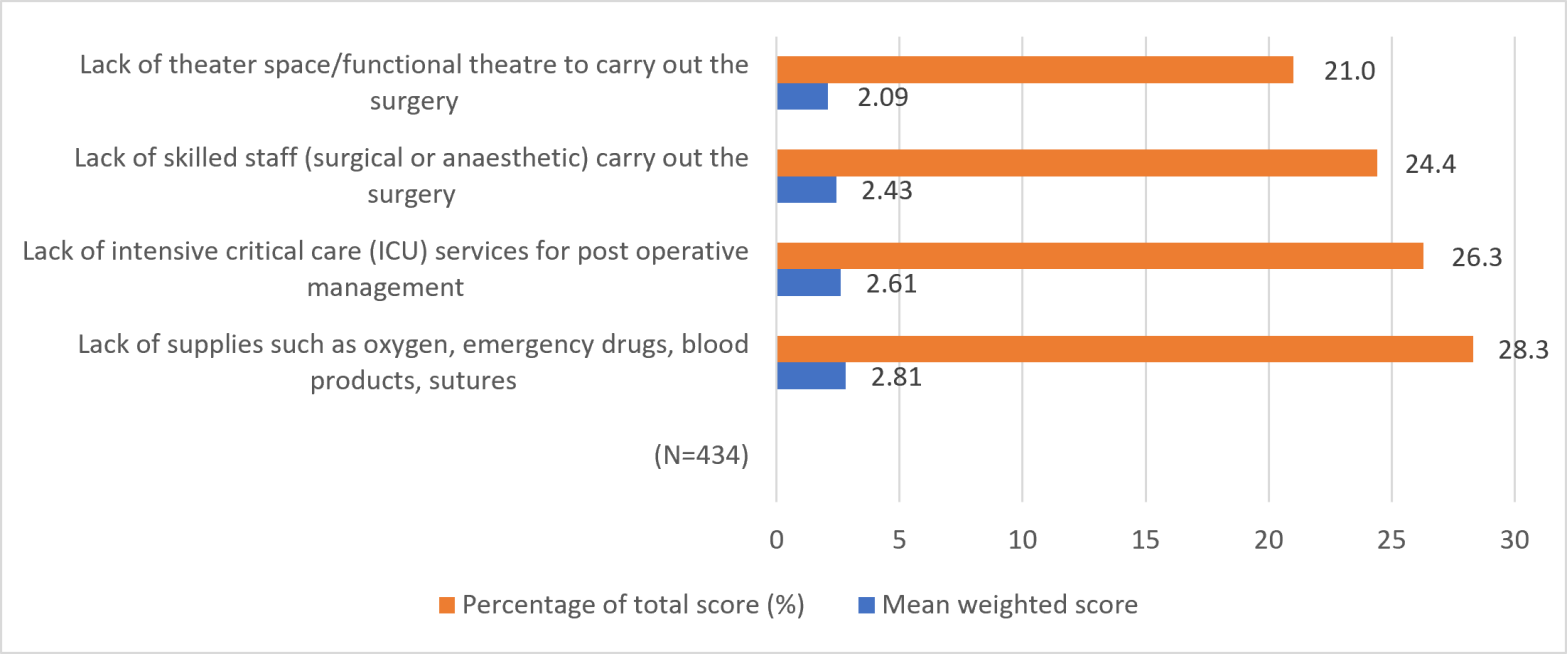
Mean weighted scores for in-hospital barriers after decision to operate (delay 3).

The highest ranked in-hospital system barriers to injury care were a shortage of emergency medical supplies (123/434, 28%) and suboptimal critical care services (114/434, 26%) (Figure 3).

Qualitative clinical audits revealed a limited range of laboratory diagnostic services available in public facilities, contributing to delays in treatment decisions. Additionally, a general shortage of essential medical and surgical supplies for both pre- and postoperative care, including pharmaceuticals and blood products, was noted.

*My role as an intern was to stabilize the patient before theatre once the decision to operate was made by the consultant. It frustrated me when I could not get the lab results and blood products on time because of having no reagents, yet my boss was on my neck. I was waiting for the relatives to bring the results from private*.

[Participant no. 133, intern doctor]

*He was supposed to get labetalol for his intracranial pressure before brain surgery; I am not sure how far his relatives have gone with lobbying for that. The orthopedic team also says he has not yet bought the implants for his fractures. There were social issues there, but I think the team will gather when the resources are available*.

[Participant no. 154, surgery resident]

**Figure 4.**
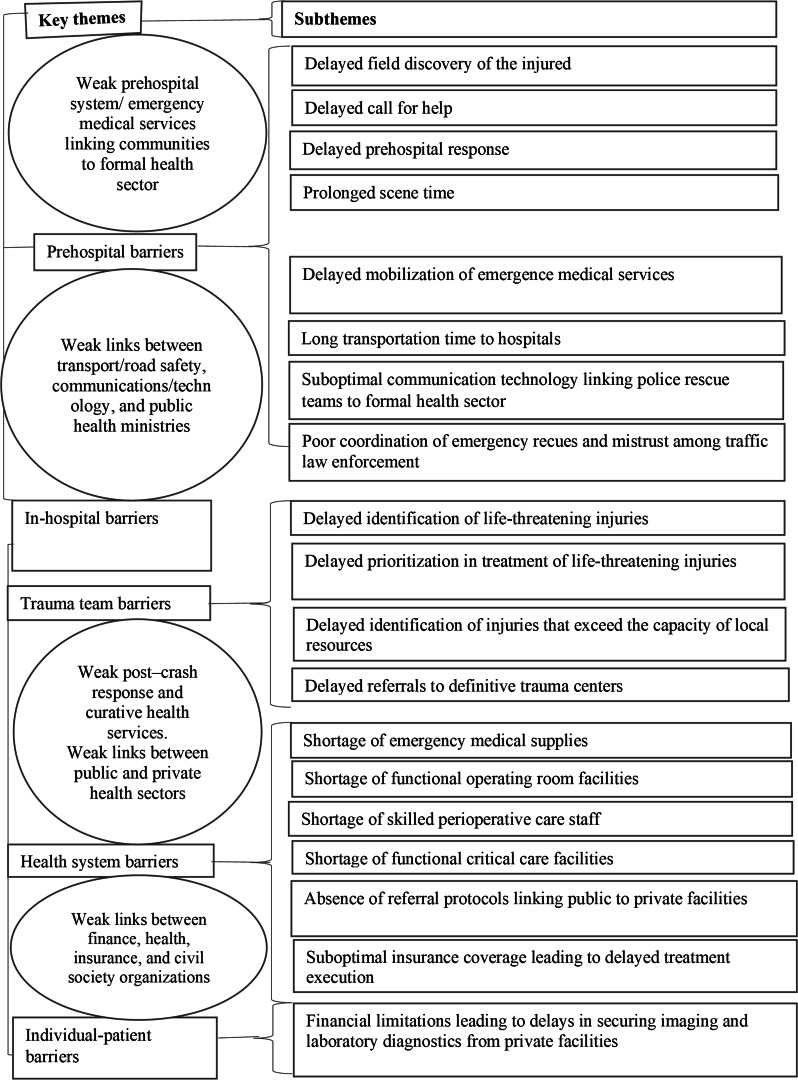
Thematic map summarizing themes and subthemes emerging from qualitative analyses of barriers to injury care.

A summary of key themes and subthemes derived from the qualitative analyses regarding barriers to injury care is presented in Figure 4.

### Quantitative Findings

#### Overview

Kruskal-Wallis test showed statistically significant differences in how the various cadres ranked the various barriers (Multimedia Appendix 3). Of the 1003 patients with trauma in the MOTOR for quantitative analysis, there were 82% (817/1003) males and 19% (186/1003) females. The overall median age (IQR) was 28 (22-37) years. The median (IQR) commute distance was 5 (2-10) km, and median (IQR) prehospital time was 2 (1-3) hours from accident scene to the emergency departments. Neurotrauma was prevalent in 95% (949/1003), whereas musculoskeletal injuries occurred in 80% (799/1003) of participants. A majority (745/1003, 74%) of participants sustained polytrauma. The median (IQR) baseline injury severity scores–based GCS and KTS were 14 (11-15) and 8 (7-9), respectively.

#### Characteristics of Patients Who Encounter Barriers to Injury Care

We analyzed 90-day complete follow-up data for 88% (887/1003) of individuals with traumatic brain injuries, and for 80% (799/1003) of individuals with musculoskeletal injuries. The characteristics of those who experienced barriers are summarized in Table 1. This group included patients who were critically ill, with 64% (272/426) presenting with a moderate to severe KTS and 45% (142/426) exhibiting moderate to severe GCS scores. Furthermore, 31% (133/426) of these individuals sustained multiple injuries that necessitated admission. For individuals with traumatic brain injuries, the proportion of unfavorable outcomes was significantly higher among those who reported barriers (84/366, 23% compared with 45/521, 9%), with *χ*^2^_1_ (N=887)=35.44 and *P*<.001. In contrast, for those with musculoskeletal injuries, there was no statistically significant difference in the proportions of unfavorable trauma outcomes between individuals who encountered barriers and those who did not (74/311, 24% vs 121/494, 25%), as indicated by *χ*^2^_1_ (N=805)=0.05 and *P*=.82. However, the mortality rate was higher among individuals who reported barriers to injury care than among those with no such barriers (55/366, 15% vs 27/521, 5%), with *χ*^2^_1_ (N=887)=24.83 and *P*<.001, as shown in Table 1.

**Table 1.** Characteristics of patients with trauma who experienced barriers to injury care.^a^

Variable	Barrier to receiving injury care reported		Total	Chi-square (*df*)	*P* value
	No	Yes			
Age (years), median (IQR)	22 (27‐35)	22 (30‐40)	28 (22‐37)	N/A^b^	<.001
Sex, n (%)				0.19 (1)	.62
Male	767 (81.6)	50 (79.4)	817 (81.5)		
Female	173 (18.4)	13 (20.6)	186 (18.5)		
Commute distance from accident scene to emergency department (km), median (IQR)	5 (2-10)	5 (2-10)	5 (2-10)	N/A	.83
Musculoskeletal injuries present?, n (%)				6.98 (1)	.008
No	134 (23.2)	70 (16.4)	204 (20.3)		
Yes	443 (76.8)	356 (83.6)	799 (79.7)		
Neurological injuries present?, n (%)				0.30 (1)	.58
No	33 (5.7)	21 (4.9)	54 (5.4)		
Yes	544 (94.3)	405 (95.1)	949 (94.6)		
Respiratory rate at admission (breaths per minute), n (%)				54.76 (2)	<.001
≤9	3 (0.7)	5 (0.9)	8 (0.8)		
10‐29	292 (70.2)	519 (88.4)	811 (80.9)		
≥30	121 (29.1)	63 (10.7)	184 (18.3)		
Systolic BP^c^ at admission (mm Hg), n (%)				21.73 (2)	<.001
≤49	2 (0.5)	0 (0)	2 (0.2)		
50‐89	100 (24.0)	79 (13.5)	179 (17.8)		
≥90	314 (75.5)	508 (86.5)	822 (82.0)		
Accident scene to emergency department admission interval (hours), median (IQR)	1.8 (1.0‐3.0)	2.0 (1.0‐3.0)	2.0 (1.0‐3.0)	N/A	.88
Referral decision to dispatch interval (hours), median (IQR)	2.5 (1.7‐3.8)	3.0 (2.0‐4.0)	2.8 (1.8‐3.9)	N/A	.78
Number of serious injuries requiring an admission, n (%)				70.84 (2)	<.001
>1	78 (13.3)	133 (34.4)	221 (22.0)		
1	390 (66.4)	233 (56.0)	623 (62.1)		
None	119 (20.3)	40 (9.6)	159 (15.9)		
KTS^d^, median (IQR)	8 (6-9)	9 (8-9)	8 (7-9)	N/A	<.001
KTS categories, n (%)				42.05 (1)	<.001
Mild (scores 9‐10)	328 (56.9)	154 (36.2)	482 (48.1)		
Moderate (scores 7‐8) or severe (score ≤6)	249 (43.1)	272 (63.9)	521 (51.91)		
GCS^e^, median (IQR)	13 (10‐15)	15 (12‐15)	14 (11‐15)	N/A	<.001
GCS categories, n (%)				24.10 (1)	<.001
Mild (scores 13‐15)	404 (70.0)	234 (54.9)	638 (63.6)		
Moderate (scores 9‐12) or severe (score ≤8)	173 (30.0)	192 (45.1)	365 (36.4)		
GOS^f^, median (IQR)	4 (4-5)	5 (4-5)	5 (4-5)	N/A	<.001
GOS categories, n (%)				35.44 (1)	<.001
Favorable outcome (scores 4‐5)	476 (91.4)	282 (77.1)	758 (85.5)		
Unfavorable outcome (scores 1‐3)	45 (8.6)	84 (23.0)	129 (14.5)		
TOMS^g^ categories, n (%)				0.05 (1)	.82
Favorable	373 (75.5)	237 (76.2)	610 (75.8)		
Unfavorable	121 (24.5)	74 (23.8)	195 (24.2)		
Injury outcome at 90 days, n (%)				24.83 (1)	<.001
Survived	494 (94.8)	311 (85.0)	805 (90.8)		
Died	27 (5.2)	55 (15.0)	82 (9.2)		

a Level of statistical significance (*P*=.05). Data are n (%) unless specified otherwise. The characteristics of all those included in analyses are demonstrated.

b Not applicable.

c BP: blood pressure.

d KTS: Kampala Trauma Score.

e GCS: Glasgow Coma Scale.

f GOS: Glasgow Outcome Scale.

g TOMS: Trauma Outcome Measure Scale score.

#### In-Hospital Audit of Nature of Common Barriers to Injury Care for the Patient Participants (Delay 3)

From 1003 audited trauma cases, only 59% (587/1003) received treatment for their injuries without their clinical teams encountering any barriers in the provision of definitive care. Hospitals whose trauma teams received rural trauma team development training identified more barriers than their counterparts (256/501, 51% vs 170/502, 34%; *P*<.001). The proportion of patients who faced 1 or more barriers was 23.1% (232/1003) at the individual (patient) level, 6.3% (63/1003) at trauma-team level, and 17.9% (180/1003) at hospital-system level. Team-level barriers referred to obstacles that resulted in delays in the activation of emergency teams, as well as in the identification, prioritization, recognition, or timely referral of life-threatening injuries. Individual-level barriers were those that impeded timely decisions regarding the initiation of treatment or the referral of injuries that exceed local capacity. In contrast, in-hospital system–level barriers were systemic obstacles that caused delays in obtaining essential supplies, such as oxygen, anesthetics, sutures, blood products, and diagnostics, as well as in securing access to theater space and intensive or critical care services.

#### Factors Associated With Health Care Access Barriers and Their Impact on Injury Outcomes

The results of factors associated with barriers to injury care are presented in Table 2. After adjusting for clustering effects by hospital teams and resources, mixed-effects regression analyses indicated that the factors independently associated with barriers to injury care included increasing age, involvement in motorcycle-car crashes, the multiplicity of injuries requiring admission, the presence of moderate or severe injuries as assessed by both the KTS and the GCS, the occurrence of axial head lesions, and the necessity for craniotomy.

Further analysis demonstrated that the presence of barriers was independently associated with unfavorable GOS scores (odds ratio [OR] 3.15, 95% CI 2.23‐4.66; *P*<.001) for neurological injuries and was linked to a 4-fold increase in odds of 90-day mortality (OR 4.20, 95% CI 2.25‐6.94; *P*<.001). However, barriers were not significantly associated with unfavorable TOMS scores for musculoskeletal injuries.

Multilevel analyses identified key factors associated with barriers, including arrival by public means (adjusted OR [aOR] 1.62, 95% CI 1.09‐2.41; *P*=.02), increasing age (aOR 1.01, 95% CI 1.00‐1.03; *P*=.01), and sustaining 1 or more injuries requiring admission (aOR 1.92, 95% CI 1.18‐3.14; *P*=.01 vs aOR 3.69, 95% CI 1.95‐6.98; *P*<.001), as well as a severe KTS score of ≤6 (aOR 2.71, 95% CI 1.37‐5.37; *P*=.004). Conversely, a negative head CT result and skull fractures were associated with lower odds of encountering barriers in both bivariate and multilevel analyses as shown in (Table 2).

**Table 2.** Multilevel mixed-effects logistic regression analyses of factors associated with barriers to injury care.^a^

Variable	Crude odds ratio	95% CI	*P* value	Adjusted odds ratio	95% CI	*P* value
Age (years)	1.018	1.008-1.027	<.001	1.014	1.003-1.025	.01
Sex						
Female	Reference	N/A^b^	N/A	N/A	N/A	N/A
Male	0.900	0.650-1.247	.53	N/A	N/A	N/A
Injury mechanism						
Motorcycle-motorcycle crash	Reference	N/A	N/A	N/A	N/A	N/A
Motorcycle-pedestrian crash	1.051	0.761-1.450	.76	N/A	N/A	N/A
Motorcycle-car crash	1.747	1.238-2.464	.001	N/A	N/A	N/A
Motorcycle-static object	0.988	0.648-1.505	.95	N/A	N/A	N/A
Commute distance from injury to emergency department (km)						
<5	Reference	N/A	N/A	N/A	N/A	N/A
>5	0.803	0.621-1.037	.09	N/A	N/A	N/A
Mode of arrival at emergency department						
By ambulance	Reference	N/A	N/A	N/A	N/A	N/A
By public taxi/motorcycle	1.120	0.813-1.545	.49	1.621	1.092-2.405	.02
By other means (walk-in)	0.367	0.133-1.015	.05	0.635	0.192-2.101	.46
Prehospital care received prior to arrival at emergency department?						
Yes	Reference	N/A	N/A	N/A	N/A	N/A
No	1.214	0.940-1.569	.14	N/A	N/A	N/A
Number of serious injuries requiring admission^c^						
None	Reference	N/A	N/A	N/A	N/A	N/A
1	2.132	1.423-3.194	<.001	1.923	1.179-3.137	.01
>1	6.285	3.948-10.007	<.001	3.693	1.954-6.980	<.001
Injury severity based on Kampala Trauma Score						
Mild (scores 9‐10)	Reference	N/A	N/A	N/A	N/A	N/A
Moderate (scores 7‐8)	1.567	1.168-2.103	.003	0.987	0.638-1.526	.95
Severe (score ≤6)	4.913	3.390-7.120	<.001	2.713	1.372-5.368	.004
Injury severity based on Glasgow Coma Scale score						
Mild (scores 13‐15)	Reference	N/A	N/A	N/A	N/A	N/A
Moderate (9-12)	1.98	1.441-2.721	<.001	1.169	0.642-2.129	.61
Severe (score ≤8)	3.166	1.99-5.027	<.001	1.330	0.607-2.915	.48
Head CT^d^ findings						
CT not indicated	Reference	N/A	N/A	N/A	N/A	N/A
Negative CT results	0.448	0.310-0.646	<.001	0.543	0.353-0.834	.01
Extra-axial lesions	1.230	0.845-1.791	.28	0.855	0.465-1.572	.62
Axial lesions	1.983	1.180-3.333	.01	0.753	0.377-1.503	.42
Skull fractures	0.421	0.249-0.711	.001	0.279	0.139-0.558	<.001
Prehospital interval (hours)						
<1	Reference	N/A	N/A	N/A	N/A	N/A
>1	1.106	0.839-1.459	.48	N/A	N/A	N/A
Referral-dispatch interval (hours)						
<1	Reference	N/A	N/A	N/A	N/A	N/A
>1	0.621	0.461-0.838	.002	N/A	N/A	N/A
Neurosurgical treatment						
Watchful waiting	Reference	N/A	N/A	N/A	N/A	N/A
Craniotomy	1.530	1.134-2.065	.01	0.718	0.358-1.437	.35
Decompressive craniectomy	1.734	0.767-3.922	.19	0.383	0.125-1.180	.10
Glasgow Outcome Scale category						
Favorable (scores 4‐5)	Reference	N/A	N/A	N/A	N/A	N/A
Unfavorable (scores 1-3)	3.151	2.231-4.658	<.001	0.543	0.267-1.107	.09
Trauma Outcome Measure Scale score						
Favorable	Reference	N/A	N/A	N/A	N/A	N/A
Unfavorable	1.071	0.737-1.555	.74	N/A	N/A	N/A
90-day injury outcomes						
Alive	Reference	N/A	N/A	N/A	N/A	N/A
Died	4.202	2.245-6.937	<.001	1.228	0.0512-2.945	.65

a Mixed-effects logistic regression integration method: mvaghermite, number of observations=861, number of treatment arms=2, average size per treatment arm=501, residual intraclass correlation for intervention vs control arm for adjusted model=0.05, 95% CI (0.01-0.31), Akaike information criterion=1037.4, and Bayesian information criterion=1127.8.

b Not applicable.

c Serious injury refers to one that warrants admission on its own merit.

d CT: computed tomography.

### Convergence

The aggregated data indicated that the components of delay 1 exhibited divergence, while convergence was observed for delays 2 and 3. A summary of the integration between qualitative and quantitative findings is presented in Table 3.

**Table 3.** A joint display summary showing the intersection of qualitative and quantitative results.

Theme/construct	Quantitative metrics	Quantitative findings	Qualitative quotes	Convergence status	Implications and overall interpretation
Delay 1: delays in decision to seek injury care	Prehospital interval in hours	Prehospital intervals of more than 1 hour were not independently associated with reported barriers to injury care (OR^a^ 1.11, 95% CI 0.84‐1.46; *P*=.48).	“You want to help evacuate these accident victims from scenes, but they think you are interrogating, arresting, and prosecuting them for their traffic crimes.” “He arrived late in a taxi as there was no fuel in the ambulance from the referring hospital.”	Divergent	Prehospital time was confounded by several factors including public mistrust, scene time, commute distance, and ambulance availability, which impacted decisions to seek injury care.
Delay 2: delays in reaching injury care	Referral decision to dispatch interval in hours	Referral to dispatch intervals of more than 1 hour were associated with lower odds of reported barriers to injury care (OR 0.62, 95% CI 0.46-0.84; *P*=.002).	“Due to understaffing we are often unable to undertake transfer responsibilities timely.” “You want to communicate the transfer timely but the poor communication technologies and network breakdown will let you down.” “Our intercom is not directly linked to hospital emergency departments.”	Convergence	Prolonged referral intervals were linked to barriers in accessing timely definitive injury care. Hospitals would delay referrals when the demands of patient care did not exceed the available local resources or when the necessary resources for executing the referrals were unavailable.
Delay 3: delays in receiving adequate injury care.	Emergency department arrival to head CT^b^ imaging as a measure of in-hospital delays	Negative CT results were associated with lower odds of reporting barriers (OR 0.54, 95% CI 0.35-0.83; *P*=.01). Conversely, severe Kampala Trauma Scores ≤6 were associated with increased odds for reporting barriers to injury care (OR 2.71, 95% CI 1.37‐5.37; *P*=.004).	“We do not have direct referral pathways to private hospitals for CT.” “The patient’s next of kin is still mobilizing financial resources to outsource CT from private.” “Our intensive care unit is full, so we plan to refer him to a private hospital, but his family is still mobilizing resources for ICU costs.”	Convergence	Critically ill patients with severe injuries face obstacles in accessing injury care such as dysfunctional referral protocols and suboptimal insurance coverage compared with less severely injured individuals.
Injury outcomes at 90 days	Injury morbidity based on proportions of unfavorable outcomes and proportions/odds of mortality	Multiplicity of injuries was associated with increased odds of encountering barriers in the continuum of injury care (OR 3.69, 95% CI 1.95‐6.98; *P*<.001). Encountering barriers was independently associated with 3-fold increase in unfavorable Glasgow Outcome Scores (OR 3.15, 95% CI 2.23‐4.66; *P*<.001) and 4-fold increase in 90-day mortality (OR 4.202, 95% CI 2.245‐6.937; *P*<.001).	“He was supposed to get labetalol for his intracranial pressure from head injury before brain surgery; the relatives need to lobby for that. The orthopedic team also says he has not yet bought the implants for his fractures. The multidisciplinary team will gather when everything is in place.” “We had to wait until patients’ relatives availed us with the results of the investigations from private before operating.”	Complementary	Multiple injuries were linked to delayed access to critical surgical care interventions. These delays could contribute to undesired injury outcomes and increased mortality.

a OR: odds ratio.

b CT: computed tomography.

## Discussion

### Principal Findings

The objective of this study was to identify critical obstacles within the continuum of trauma care, both before and during hospital admission, as perceived by first responders. We found that there was a convergence of key themes between those reported by frontline health workers during the qualitative interviews and those elicited by the attending clinicians during the real-time execution of patient treatment in the quantitative trauma registry audit. Our results indicated notable systemic inefficiencies in the activation of emergency medical services and the arrival of ambulances, which may exacerbate the severity of injuries and negatively impact patient outcomes. Moreover, a lack of designated rural networks for emergency responses, as highlighted by the law enforcement officials, exacerbates the situation in remote areas. Furthermore, the absence of clear protocols for traffic crash victim evacuation may negatively impact the quality of care provided. Similar impediments have been earlier identified in remote settings of other LMICs, highlighting the need to strengthen rural trauma systems [20,38].

Qualitative analyses uncovered organizational and system-level factors encountered by law enforcement and referring clinicians. For instance, understaffing and the absence of clearly defined roles for emergency road traffic response personnel underscore the necessity for strategic workforce management and explicit assignment of responsibilities. The complex interplay among understaffing, role ambiguity, and task delays driven by burnouts due to heavy workload degrades emergency care processes. These effects reflect a mismatch between people, tasks, and tools, which collectively undermines the health system’s ability to deliver safe, high-quality care. Within the Systems Engineering Initiative for Patient Safety, these workforce-related conditions operate as work system determinants that shape how care tasks are allocated, coordinated, and executed.

Moreover, there were reported inefficient communication technologies that impeded effective trauma coordination between emergency responders and recipient hospitals, delaying patient transfers. Research shows that such inefficiencies could result in the loss of critical information and compromise patient care [13]. In a recent scoping review of handover practices between paramedics, emergency department staff, and trauma teams, lost information due to communication gaps was associated with diagnostic errors, missed injuries, and increased mortality [39].

Furthermore, we identified psychosocial constraints to an effective emergency response framework manifested by public bias and mistrust of law enforcement trauma evacuation teams. This mistrust led to withholding valuable information regarding the circumstances of injury, which would be essential for assessing the liability and settlement of insurance claims, thereby reducing out-of-pocket expenditure. These observations reinforce the need for comprehensive community engagement and awareness campaigns to build public trust. Statutorily integrating police services with health care systems may bridge these gaps [40]. However, this unified approach requires clearly defined dispatch protocols with explicit role specifications, structured assessment checklists, and quality safeguards with clinical oversight of scope of practice to ensure patient safety and compliance with data-protection policies.

We found in-hospital barriers characterized by a shortage of surgical supplies, which delayed the identification and treatment of life-threatening injuries. Access to essential medical supplies and a competent health workforce are an integral component of World Health Organization (WHO) building blocks for resilient health systems [41]. A sustainable robust logistical framework is mandatory to ensure that clinicians can quickly assess, diagnose, and treat life-threatening conditions. In quantitative analyses, the plausible correlation between the presence of barriers, injury severity, and unfavorable outcomes further emphasizes that these systemic issues not only hinder immediate care but also have lasting implications for the recovery and survival of very critically ill patients whose care demands exceed local resources. The increased odds of mortality among those who encounter obstacles highlight the urgent need for interventions aimed at streamlining care processes and resource allocation within trauma care systems. Comparable research conducted in Rwanda, Ghana, and South Africa assessed the application of multiple data collection methods to identify context-specific blockages to injury care that contributed to mortality and disability, and our findings are consistent with these studies [38].

Finally, the obstacles identified by the frontline workers during the real-time execution of treatment, such as inadequate diagnostic resources, transportation failure, and financial difficulties, spotlight systemic challenges within the Ugandan health care sector. Sustainable health care financing is an additional core WHO building block because it protects patients from catastrophic expenditures [41]. In a recent Delphi study conducted in Malawi [42], the absence of readily available physical resources emerged as the most significant factor contributing to in-hospital delays. Moreover, the high financial cost of care and the unaffordability of emergency transport were identified as the primary factors leading to prehospital delays [42]. These challenges reflect inadequate resource allocation to trauma care and to the broader health sector, which has negatively impacted the quality of health systems in both LMICs and HICs [43,44]. For instance, a systematic review of barriers to injury care in 21 American countries revealed that a lack of resources and equipment constituted 58% (33/57) of barriers, followed by the absence of protocols, understaffing, transport and logistics challenges, and financial limitations [45]. Further research from Australia and Europe elaborates more on these challenges, highlighting rural-urban and socioeconomic disparities amid declining social support structures [46,47].

Poor-quality health care and inadequate financial risk protection are now considered the major drivers of mortality, surpassing inadequate health access, which has delayed the achievement of SDG3 targets 3.6 and 3.8 in vulnerable populations, irrespective of country-specific development index [43,48]. Furthermore, the reported inefficient communication technologies that impede effective trauma coordination, coupled with inadequate infrastructural capabilities influenced by social factors in health systems, could impede the attainment of SDG9 [48]. Thus, attaining universal health coverage should not be regarded as a stand-alone but rather a multisectoral initiative that incorporates elements of SDG9. This would foster technological innovations for medical equipment, build inclusive infrastructure such as safe road networks and accessible hospitals with functional emergency departments, and establish resilient industries that foster research development and equitable medical education to address low-quality and health access barriers [49]. Our results indicate weak connections between the formal and informal sectors, public and private sectors, and health and technology sectors. Addressing these gaps through strong advocacy by civil society organizations could meet the care demands of severely injured patients and enhance the overall learning and work environment for trainee physicians in low-resource settings, thereby improving the quality of their medical education (SDG 4).

### Policy Implications

Our study has identified multiple significant obstacles present at the various stages of the trauma care continuum. Most of these identified challenges are applicable to other LMICs [20,38,42]. Consequently, the hindrances to injury care from the perspectives of trainee physicians and traffic law enforcement first responders must be addressed to accurately conceptualize their expected roles in the trauma care continuum and patients’ advocacy. Addressing these barriers requires multidisciplinary approaches focusing on quality prehospital response, swift safe transportation, and robust postcrash care systems. Stakeholders, including the National Road Safety Council, Ugandan Ministry of Health, Ministry of Education, and Regional Police Commanders, should engage in planning meetings to address these critical issues.

All the financial, logistical, and system-level impediments to trauma care elicited in this study are direct obstacles to the WHO health systems building blocks [41]. This study provides context for the implementation strategies needed to address gaps in injury care. In Uganda, policymakers should strengthen the existing Integrated Disease Surveillance and Response system by adapting monitoring tools including electronic logistics management and health information systems to track stockouts of essential medical supplies, human resources observatories to monitor health workforce distribution and service delivery inequities, and national health accounts to monitor health financing, public expenditure, and financial risk protection. These measures, aligned with WHO guidance and the Global Health Observatory, would form a coherent framework for assessing and improving health sector performance regarding trauma care [41].

Regarding individual-patient and trauma-team delays, the interplay between limited insurance coverage and insufficient disposable incomes leads to substantial delays as caretakers mobilize funds for critical investigations and emergency surgical procedures. For instance, a brain computed tomographic scan can cost a substantial amount of up to US $130, commensurate to the average monthly salary of green-collar workers in Uganda [24]. Consequently, trauma teams delay surgical decisions, which may preclude high-quality surgical outcomes amidst potential threat for medical-legal litigation, as patients and families increasingly assert their autonomy in treatment decisions. The likelihood that individuals with severe injuries have high care demands, which may exceed local resources, necessitates advocacy for improvement in the health system to adequately address these needs. To achieve this goal, stewardship that promotes good governance and team leadership, together with routine community health insurance engagement, should accompany health system performance assessments in line with the WHO building blocks for sustainable health systems.

In-hospital system delays in injury care were primarily attributed to a lack of supplies and critical care services, with these constraints ranking second in an audit of patient handling. Key supplies such as oxygen, anesthetics, and blood products, as well as access to operating theaters and intensive care units, are all mandatory requisites for the survival of patients with major trauma. Access to good-quality service delivery such as critical care underpins one of the WHO building blocks. Previous studies indicate that dual deficiencies in both equipment and human resources imply a severe hindrance to lifesaving interventions, contributing to a substantial proportion of preventable trauma deaths in LMICs [13,20]. To mitigate these adversities, investment in reliable supply chain management through private-public partnerships, improving ambulance coordination through sufficient staffing, and establishing financial support systems for uninsured patients could streamline referral pathways to ensure continuity of care in emergency situations. The use of noncontributory funding sources generated through public taxation, pooling funds across multiple public sectors, and strong accountability to track performance of the designated funds could mitigate the shortage of supplies and safeguard patients against catastrophic expenditures [48].

### Strengths and Limitations

The strengths of this study dwell in its mixed methods approach, which enabled a nuanced analysis of barriers encountered at various stages of real-world injury care. Additionally, by focusing on the perspectives of frontline workers directly involved in trauma care, the study provides valuable insights into context-specific challenges encountered in LMIC settings. Moreover, the study contributes valuable evidence on the intersection of trauma systems, medical education, and health equity in low-resource settings, which is relevant to the attainment of SDGs 3 and 4.

However, there were limitations that should be recognized. First, the use of Likert scales may pose challenges for interpretation although they were applied to mixed methods to enrich qualitative inquiry and facilitate triangulation with quantitative data. While the study used parametric tests to enhance statistical power, this approach might not align with best practices for exploratory research that lacks prior hypotheses [50].

Second, the study assumes that accumulated Likert items can serve as a reasonable surrogate for continuous interval measurements. Although this assumption facilitates the computation of overall mean scores to enable objective ranking for the barriers, it may potentially compromise the precision of central tendency measures [51]. Moreover, while we endeavored to interpret the data objectively through triangulation and use of parametric tests, we acknowledge the possibility of bias arising from the integration of diverse data paradigms which used varying collection and sampling methods. This concern reflects a significant and ongoing debate within the field of mixed methods research [52].

Third, the reliance on data saturation during interviews may have unintentionally constrained the identified barriers to dominant themes, thereby silencing divergent minority opinions. This represents an inherent methodological issue in qualitative research, which often prioritizes the depth and richness of data over numerical targets [31]. Moreover, we acknowledge that recall in qualitative responses and the missing 90-day outcome data may have introduced systematic bias.

Finally, we acknowledge that aggregating injury severity into subcategories of mild, moderate, and severe, and outcomes as favorable versus unfavorable, may lead to a loss of information, contributing to residual confounding in assessing the true impact of barriers on injury outcomes. Moreover, in view of other host and environmental factors that impact injury outcomes but were not assessed in this study, patients with less severe injuries may have had minimal care needs, which could prevent attending clinicians from identifying significant in-hospital barriers during their treatment, compared with the severely injured patients, an issue that future studies should address. Nevertheless, the study assumed that stratifying trauma outcomes by injury severity categories would minimize this confounding.

### Conclusions

This mixed methods study evaluated the key barriers to injury care within the context of frontline health workers in low-resource settings. We identified significant barriers in the prehospital and in-hospital trauma care continuum, which could adversely impact patient outcomes. These systemic challenges require multifaceted targeted interventions that integrate operational improvements to address system-level factors and logistical frameworks through community engagement. The results of this study warrant an urgent need to secure sustainable hospital supplies and establish clear referral protocols that link the public to private facilities. These interventions would enable timely treatment decisions to optimize trauma care delivery and treatment outcomes, especially for those who sustain severe injuries. Future research should be robust multicenter prospective cohort studies that integrate mixed methods to investigate how these barriers impact long-term injury outcomes in diverse socioeconomic settings.

## Supplementary material

10.2196/84774 Multimedia Appendix 1 Barriers to injury care identified through qualitative structured interviews and focus groups.

10.2196/84774 Multimedia Appendix 2 Motorcycle trauma and outcome registry data sheet.

10.2196/84774 Multimedia Appendix 3 Mean rank scores for different barriers to injury care by the various cadres.

10.2196/84774 Checklist 1 COREQ checklist.

10.2196/84774 Checklist 2 GRAMMS checklist.

## Abbreviations

aOR: adjusted odds ratio
COREQ: Consolidated Criteria for Reporting Qualitative Research
GCS: Glasgow Coma Scale
GOS: Glasgow Outcome Scale
HIC: high-income country
KTS: Kampala Trauma Score
LMIC: low- and middle-income country
MOTOR: Motorcycle Trauma Outcome Registry
OR: odds ratio
REDCap: Research Electronic Data Capture
SDG: Sustainable Development Goal
TOMS: Trauma Outcome Measure Scale
WHO: World Health Organization
