# Supplementary material for: Understanding Barriers to Effective Injury Care by Medical Trainees and Traffic Law Enforcement First Responders in Low-Income Contexts in Uganda (Motor Registry Project Part 2): Convergent Mixed Methods Analysis
Source: JMIR Hum Factors. 2026 Jun 10;13:e84774. doi: 10.2196/84774 (PMC13252699; doi:10.2196/84774)
Supplement: Multimedia Appendix 1 [file humanfactors-v13-e84774-s001.docx]

Multimedia Appendix 1. Barriers to injury care identified through qualitative structured interviews and focus groups.

**Part 1: Individual structured interview**

Introduction: Please rank the following identified delays as barriers to timely provision of definitive injury care for trauma patients in general.

Instructions: For each question, please assign each numeric number once.

1. In your current hospital of attachment, please rank the potential significant **system delays** to providing care to the injured (trauma) patients, in order of importance from the most important (score of 6) to the least important (score of 1) by assigning numbers 1, 2, 3, 4, 5, 6
2. Delays due to Discovery of the injured in the field
3. Delays in summoning/calling for help
4. Delays in mobilizing emergency medical service (EMS)/ambulance providers
5. Delays due to pre-hospital response time (ambulance arrival to scene)
6. Prolonged scene time (Delay at the scene)
7. Long transportation time to the hospital
8. Other (specify)……………………………………………………………………………
9. After a trauma patient has arrived at your current hospital of attachment, please rank the potential significant **team delays** to providing care to such patient in order of importance (by assigning a score of 4 to the most significant and 1 to the least significant (use 1,2,3,4)
10. Delays in identification of immediately life-threatening injuries
11. Delays in prioritization of life-threatening injuries
12. Delays in recognition of injury severity
13. Delays in transportation to a higher level of care
14. Other (specify)…………………………………………………………………………
15. In your current hospital, after the decision has been made to carry out emergency surgery for a trauma patient (for example amputation for a crushed limb), please rank the potential significant **barriers** to providing surgery to such patient in order of importance (by assigning a score of 4 to the most significant and 1 to the least significant. (use numbers 1,2,3,4)
16. Lack of skilled staff (surgical or anesthetic) to carry out the surgery
17. Lack of theatre space/functional theatre to carry out the surgery
18. Lack of supplies such as oxygen, emergency drugs, blood products, sutures
19. Lack of intensive and critical care services for post-operative management

Other (specify)……………………………………………………………… …………

**Part 2: Focus groups**

Introductions: You are a mixed group of 5 individuals with varied background e.g., traffic law enforcement professionals, third-year medical student (junior clerk), fifth-year medical student (senior clerk), surgery intern doctor or intern nurse (junior house officer), Master of Surgery resident (senior house officer).

Instructions: Based on your own experiences and based on what you have observed at your hospital or workstation/work environment, provide context to each of the following regarding how it constitutes as a barrier to provision of definitive injury care for trauma patients. Please justify your opinion by mentioning real-world examples you have encountered. You may also comment on how your team members’ experiences differed or were like your own by providing your own context. Your responses will be recorded for clarity and follow-up questions.

1. Issues before patients reach your hospital e.g., before police or ambulance drops them to casualty.
2. Delays due to discovery of the injured persons in the field ………………………………..
3. Delays in summoning/calling for help for the injured persons ……………………………..
4. Delays in mobilizing emergency medical service (EMS)/ambulance providers ……………
5. Delays due to pre-hospital response time (ambulance arrival to accident scene) …………
6. Prolonged scene time (delay at the accident scene) ………………………………………
7. Long transportation time to the hospital ……………………………………………………
8. Other (specify e.g., connectivity issues, failed referrals during coordinating transfers……
9. Issues from the treating team (s) after a trauma patient has arrived at your current hospital
10. Delays in identification of immediately life-threatening injuries…………………………
11. Delays in prioritization of life-threatening injuries ………………………………………
12. Delays in recognition of injury severity ………………………………………………….
13. Delays in transportation to a higher level of care ………………………………………
14. Other (specify e.g., delayed decisions to admit, operate etc)…………………………….
15. Issues after the patient is already admitted in the hospital e.g., after decision to carry out an emergency surgery for a trauma patient has been made by your senior.
16. Lack of skilled staff (surgical or anesthetic or nursing or support) to carry out the surgery…...
17. Lack of theatre space/functional theatre to carry out the surgery ……………………………
18. Lack of supplies such as oxygen, emergency drugs, blood products, sutures ……………...
19. Lack of intensive and critical care services for pre or post-operative management …………

Other (specify e.g., imaging diagnostics, laboratory etc. )…………………………………………

1. What in your opinion has not been covered regarding barriers to proving injury care?..........
