# Supplementary material for: Understanding Barriers to Effective Injury Care by Medical Trainees and Traffic Law Enforcement First Responders in Low-Income Contexts in Uganda (Motor Registry Project Part 2): Convergent Mixed Methods Analysis
Source: JMIR Hum Factors. 2026 Jun 10;13:e84774. doi: 10.2196/84774 (PMC13252699; doi:10.2196/84774)
Supplement: Multimedia Appendix 2 [file humanfactors-v13-e84774-s002.docx]

Multimedia Appendix 2. Motorcycle trauma and outcome registry data sheet.

**Instructions: To be administered by the investigator or representative.** The interviewer should **tick ONLY ONE** most appropriate option and specify where necessary.

Name of the interviewer………………………….......... Sign …………….. Date ……….…….

Hospital name…………………………………………………………………………………….

**Disclosure:** Dear respondent, you are willingly requested to participate in this study and you are free to decline your participation at any time without any implications on your entitlements. The information that you provide will remain confidential.

**PART ONE: PARTICIPANTS’ DEMOGRAPHIC DATA**

Participants’ Name (Optional): ………………………………Unique Number…………………

Phone No (1).…………………………… (2)Alternative contact (next of keen)…………………

1. Age (years)……………………………………………………………………………………..

2. Sex; i) M ii) F

3. Religious Affiliation ………………………………………………………………………

4. Education level ……………………………………………………………………………

5. Marital status i) Single ii) Married iii) Divorced Other……………

6. Occupation) …………………………………………………………………………………….

7. Employment status i) Formal employment ii) Self-employed

iii) Unemployed iv) Student other………………………………………….

8. History of alcohol consumption i) Yes ii) No

9. Referral status i) Referred from a lower facility ii) Self referral

10. Estimated distance (Kilometers) from home to the health facility………………………

**PART TWO: MECHANISM AND NATURE OF INJURIES^1^**

11. Road user category i) Passenger iii) Pedestrian iii) Motorcyclist

12. Mechanism of injury i) Motorcycle-motorcycle ii) Motorcycle-pedestrian

iii) Motorcycle-car crash iv) other…………………………………………………

13. Mode of arrival i) By ambulance ii) By taxi/motorcycle iii) other…………….......

14. Estimated time lag from accident to arrival in hours………………………………………….

15. Pre-hospital care/First aid given before arrival? i) Yes No

16. If yes, who administered the first aid? ii) Health worker ii) Lay-bystander/police

17. Evidence of chronic medical illness i) Yes ii) No If yes specify … …………

18. Body system injured: i) Head ii) Neck iii) Chest

iv) Abdomen v) Pelvis vi) Musculoskeletal

19. If fracture of the tibia is present classify i) Open ii) Closed

20. Pattern of tibial fracture involvement i) Proximal 1/3 ii) Mid 1/3 iii) Distal 1/3

^1^Lule, H., Mugerwa, M., Ssebuufu, R., Kyamanywa, P., Bärnighausen, T., Posti, J. P., & Wilson, M. L. (2024). Effect of Rural Trauma Team Development on the Outcomes of Motorcycle Accident-Related Injuries (Motor Registry Project): Protocol for a Multicenter Cluster Randomized Controlled Trial. *JMIR research protocols*, *13*, e55297. <https://doi.org/10.2196/55297>

**PART 3: INJURY SEVERITY SCORES AND TREATMENT**

21. If Head injury is present/suspected, indicate total Glasgow Coma Score……………………...

22. Glasgow coma score category i) mild (13-15) ii) moderate (12-9) iii) severe≤8

| Best response | Description | Score | Tick appropriate |
| --- | --- | --- | --- |
| Eye-opening | Spontaneous | 4 |  |
|  | To verbal stimuli | 3 |  |
|  | To pain | 2 |  |
|  | No response | 1 |  |
| Verbal | Oriented | 5 |  |
|  | Confused conversation | 4 |  |
|  | Inappropriate words | 3 |  |
|  | Incomprehensible speech | 2 |  |
|  | No response | 1 |  |
| Motor | Obeys command | 6 |  |
|  | Localises pain | 5 |  |
|  | Withdraws from pain | 4 |  |
|  | Flexion in response to pain | 3 |  |
|  | Extension in response to pain | 2 |  |
|  | No response | 1 |  |
| Teasdale, G., & Jennett, B. (1974). Assessment of coma and impaired consciousness. A practical scale. *Lancet (London, England)*, *2*(7872), 81–84. <https://doi.org/10.1016/s0140-6736(74)91639-0> | | | |

23. Severity of other injuries e.g., musculoskeletal: *Kampala Trauma Score II value…….

24. Kampala trauma score category i) Mild 9-10 ii) Moderate 7-8 iii) Severe ≤ 6

^*^The Kampala Trauma Score II (Total=A+B+C+D); (Adapted from Mutooro *et al*, 2010)

| **Category** | **Clinical Parameters** | **Description** | **Score** |
| --- | --- | --- | --- |
| A | Age (Years) | 5-55 | 1 |
|  |  | <5>55 | 0 |
| B | Systolic BP on admission | >89mmhg | 2 |
|  |  | 89-50 | 1 |
|  |  | <=49 | 0 |
| C | Respiratory rate on admission | 10-29bpm | 2 |
|  |  | >=30 | 1 |
|  |  | <=9 | 0 |
| D | Neurological status | A | 3 |
|  |  | V | 2 |
|  |  | P | 1 |
|  |  | U | 0 |
| E | Score for serious injuries | None | 2 |
|  |  | One | 1 |
|  |  | >one | 0 |
| Mutooro S.M, Mutakooha E, Kyamanywa. P. (2010). A Comparison of Kampala Trauma Score II with the New Injury Severity Score in Mbarara University Teaching Hospital in Uganda. *East and Central African Journal of Surgery*, *15*(1), 62–71. | | | |

25. Decision on mode of treatment: i) Conservative ii) Operative e.g., ORIF

**PART 4: BARRIERS TO INJURY CARE AND INJURY OUTCOMES**

26. Was any barrier encountered during execution of definitive injury care i)No ii) Yes

27. Specify any barrier(s) to receiving definitive injury care encountered or documented for this particular patient as below: Please keep any additional information for follow-up audit.

i. No barrier encountered in execution of definitive injury care

ii. Team barriers (leading to delays in emergency skilled team activation, identification, prioritization, recognition, timely referral of life threatening injuries)

iii. Individual barriers (leading to delays in decision to operate, initiate treatment, referral of injuries exceeding local capacity)

iv. In-hospital system barriers (leading to delays in securing necessary supplies eg oxygen, anesthetics, sutures, blood products, functional diagnostics, theatre space, intensive and critical care services)

28. Injury outcome within 12 weeks i) Survived ii) Died

**PART 5: TRAUMA EXPECTATION FACTOR SCORE (TEFS)**

**To be administered by the investigator or representative after surgical intervention before discharge**

| SN | Item | Score (Tick as appropriate) | | | | |
| --- | --- | --- | --- | --- | --- | --- |
| 1 | 3 months (12 weeks) after surgery, how painful do you expect your injury to be? | 0 (No pain) | 1(mild pain) | 2 (moderate pain) | 3 (severe pain) | 4 (Unbearable pain) |
| 2 | 3 months (12 weeks) after surgery, how much do you expect your injury to interfere with your normal/usual necessary activity (including prolonged standing, walking, stairs, car driving, and sleeping)? | 0 (Not at all) | 1 | 2 | 3 | 4 (Completely) |
| 3 | 3 months (12 weeks) after surgery, how much do you expect your injury to interfere with your normal/usual physical activity (including work, housework, school, and recreation/sports activities)? | 0 (Not at all) | 1 | 2 | 3 | 4(completely) |
| 4 | 3 months (12 weeks) after surgery, how much do you expect your injury to interfere with your normal/usual activities of daily living (including eating, dressing, putting on shoe wear)? | 0 (Not at all) | 1 | 2 | 3 | 4(completely) |
| 5 | 3 months (12 weeks) after surgery, how much do you expect your injury to interfere with your normal/usual relationships (including family, friends, and coworkers)? | 0 (Not at all) | 1 | 2 | 3 | 4(completely) |
| 6 | Necessary activities (you have to do these).  3 months (12 weeks) after surgery, how much do you expect to cut down on the physical activities you have to do (including work, housework, and school)? | 0 (0%) | 1(25%) | 2(50%) | 3(75%) | 4(100%) |
| 7 | Optional activities (you enjoy to do these).  3 months (12 weeks) after surgery, how much to you expect to cut down on the physical activities you enjoy doing (including sports, recreation, gardening, etc.)? | 0 (0%) | 1(25%) | 2(50%) | 3(75%) | 4(100%) |
| 8 | 3 months (12 weeks) after surgery, how satisfied do you expect to be with your pain, physical function, and disability? | 0 (Not satisfied) | 1 | 2 | 3 | 4(very satisfied) |
| 9 | 3 months (12 weeks) after surgery, how satisfied do you expect to be with the appearance of your injury? | 0(Not satisfied | 1 | 2 | 3 | 4(very satisfied) |
| 10 | 3 months (12 weeks) after surgery, how satisfied do you expect to be with the appearance of your injury? | 0(Not satisfied) | 1 | 2 | 3 | 4(very satisfied) |

Modification of: Suk, M., Daigl, M., Buckley, R. E., Paccola, C. A., Lorich, D. G., Helfet, D. L., & Hanson, B. (2013). TEFTOM: A Promising General Trauma Expectation/Outcome Measure-Results of a Validation Study on Pan-American Ankle and Distal Tibia Trauma Patients. *ISRN Orthop*, *2013*, 801784. <https://doi.org/10.1155/2013/801784>

**PART 6: TRAUMA OUTCOME MEASURE SCORE (TOMS)**

**To be administered by the investigator at 3 months follow-up in outpatient or phone interview**

| SN | Item | Score (Tick as appropriate) | | | | |
| --- | --- | --- | --- | --- | --- | --- |
| 1 | How painful is your injury today? | 0 (No pain) | 1 | 2 | 3 | 4 (Unbearable pain) |
| 2 | How much does your injury currently interfere with your normal/usual necessary activity (including prolonged standing, walking, stairs, car driving, and sleeping)? | 0 (Not at all) | 1 | 2 | 3 | 4 (Completely) |
| 3 | How much does your injury currently interfere with your normal/usual physical activity (including work, housework, school, and recreation/sports activities)? | 0 (Not at all) | 1 | 2 | 3 | 4(completely) |
| 4 | How much does your injury currently interfere with your normal/usual activities of daily living (including eating, dressing, putting on shoe wear)? | 0 (Not at all) | 1 | 2 | 3 | 4(completely) |
| 5 | How much does your injury currently interfere with your normal/usual relationships (including family, friends, and coworkers)? | 0 (Not at all) | 1 | 2 | 3 | 4(completely) |
| 6 | Necessary activities (you have to do these). How much do you currently cut down on the physical activities you have to do (including work, housework, and school)? | 0 (0%) | 1(25%) | 2(50%) | 3(75%) | 4(100%) |
| 7 | Optional activities (you enjoy to do these). How much do you currently cut down on the physical activities you enjoy doing (including sports, recreation, and gardening)? | 0 (0%) | 1(25%) | 2(50%) | 3(75%) | 4(100%) |
| 8 | How satisfied are you with your current level of pain, physical function, and disability? | 0 (Not satisfied) | 1 | 2 | 3 | 4(very satisfied) |
| 9 | How satisfied are you with the current appearance of your injury? | 0(Not satisfied | 1 | 2 | 3 | 4(very satisfied) |
| 10 | How satisfied are you with your current overall well-being? | 0(Not satisfied) | 1 | 2 | 3 | 4(very satisfied) |

*Trauma expectation factor and outcome measure has been shown to have internal validity, consistence and reproducibility. The tables are modifications of (Suk et al, 2013)

Suk, M., Daigl, M., Buckley, R. E., Paccola, C. A., Lorich, D. G., Helfet, D. L., & Hanson, B. (2013). TEFTOM: A Promising General Trauma Expectation/Outcome Measure-Results of a Validation Study on Pan-American Ankle and Distal Tibia Trauma Patients. *ISRN Orthop*, *2013*, 801784. <https://doi.org/10.1155/2013/801784>

**PART 7:** **GLASGOW OUTCOME SCALE***

**To be administered by the investigator or representative at 3-months follow-up to poly-trauma patients who concurrently sustained tibial fractures and head injuries. Tick the appropriate fate of the patient after 3 months**

| Score | Parameter | Description | Tick |
| --- | --- | --- | --- |
| 1 | Death | Clinically confirmed death |  |
| 2 | Persistent vegetative state | severe damage with prolonged state of unresponsiveness and a lack of higher mental functions |  |
| 3 | Severe disability | Severe injury with permanent need for help with daily living |  |
| 4 | Moderate disability | No need for assistance in everyday life, employment is possible but may require special equipment |  |
| 5 | Good recovery | Minimal injury with minor neurological and psychological deficits. Patient is independent and employable |  |

*The Glasgow outcome scale (GOS) is a quick to administer widely accepted tool that was designed to objectively describe the degree of recovery for patients who suffered brain injuries to predict short and long term course of rehabilitation to return to work and everyday life. It has been recognized for its higher inter rater reliability, sensitivity to change, and its outcome categories that can be dichotomized (Jennett et al, 1981).

Jennett, B., Snoek, J., R Bond, M., & Brooks, N. (1981). *Jennett B, Snoeck J, Bond MR, et al: Disability after severe head injury: Observations on the use of Glasgow Outcome Scale. J Neurol Neurosurg Psychiatry 44: 285-293*. *Journal of neurology, neurosurgery, and psychiatry* (Vol. 44). https://doi.org/10.1136/jnnp.44.4.285
