# Supplementary material for: Understanding Barriers to Effective Injury Care by Medical Trainees and Traffic Law Enforcement First Responders in Low-Income Contexts in Uganda (Motor Registry Project Part 2): Convergent Mixed Methods Analysis
Source: JMIR Hum Factors. 2026 Jun 10;13:e84774. doi: 10.2196/84774 (PMC13252699; doi:10.2196/84774)
Supplement: Multimedia Appendix 3 [file humanfactors-v13-e84774-s003.docx]

Multimedia Appendix 3. Mean rank scores for different barriers to injury care by the various cadres.

|  | Kruskal-Wallis rank sum test (H) for various cadres | | | | | | |
| --- | --- | --- | --- | --- | --- | --- | --- |
| Variable | Intern Doctor (n=30) | Fifth year medical student (n=140) | Third year medical student (n=264) | Traffic Police Officer  (n=66) | Chi-square (χ^2^) | Df | P-value |
| Delay 1: Pre-hospital barriers (N=500) | | | | | | | |
| Delays in mobilizing emergency medical services/ambulance providers | 10718.0 | 33812.0 | 59390.0 | 21330.0 | 44.90 | 3 | 0.0001 |
| Delays due to pre-hospital response time (ambulance arrival to scene) | 9863.0 | 32274.0 | 58757.0 | 24356.0 | 68.93 | 3 | 0.0001 |
| Long transportation time to the hospital | 8774.5 | 37666.5 | 62864.5 | 15944.5 | 7.26 | 3 | 0.0642 |
| Prolonged scene time (delays at the scene) | 2903.0 | 3674.3 | 67955.0 | 17649.0 | 37.58 | 3 | 0.0001 |
| Delays due to discovery of the injured in the field | 7338.5 | 34176.0 | 72764.5 | 10771.0 | 34.34 | 3 | 0.0001 |
| Delays in summoning/calling for help | 5955.5 | 35916.5 | 74623.0 | 8755.0 | 63.64 | 3 | 0.0001 |
| Delay 2: In-hospital team barriers (N=434) | | | | | | | |
| Delays in identification of life-threatening injuries | 5868.5 | 27686.0 | 60840.5 | - | 7.79 | 2 | 0.0204 |
| Delays in transportation to a higher level | 10037.0 | 30834.0 | 53524.0 | - | 33.86 | 2 | 0.0001 |
| Delays in prioritization of lite-threatening injuries | 4230.0 | 30329.0 | 59835.0 | - | 14.10 | 2 | 0.0009 |
| Delays in recognition of injury severity | 3755.5 | 31874.1 | 58765.5 | - | 19.19 | 2 | 0.0001 |
| Delay 3: In-hospital delays after decision to operate (infrastructural and human resource-related delays) (N=434) | | | | | | | |
| Lack of supplies such as oxygen, emergency drugs, blood products, sutures | 10780.0 | 31481.0 | 52134.0 | - | 49.52 | 2 | 0.0001 |
| Lack of intensive critical care (ICU) services for post-operative management | 3308.5 | 29593.0 | 61493.5 | - | 29.06 | 2 | 0.0001 |
| Lack of skilled staff (surgical or anesthetic) to carry out the surgery | 6816.5 | 28766.5 | 58812.0 | - | 2.08 | 2 | 0.3533 |
| Lack of theatre space/functional theatre to carry out the surgery | 7707.0 | 31252.0 | 55436.0 | - | 4.62 | 2 | 0.0995 |
