# Supplementary material for: Understanding Barriers to Effective Injury Care by Medical Trainees and Traffic Law Enforcement First Responders in Low-Income Contexts in Uganda (Motor Registry Project Part 2): Convergent Mixed Methods Analysis
Source: JMIR Hum Factors. 2026 Jun 10;13:e84774. doi: 10.2196/84774 (PMC13252699; doi:10.2196/84774)
Supplement: Checklist 2 [file humanfactors-v13-e84774-s005.pdf]

**Good reporting of a mixed methods study (GRAMMS) checklist**[ⱡ]	**Section: (Page)**
Describe the justification for using a mixed methods approach to the research question	Introduction: pg 3<br>Study design: pg 4<br>Data collection: pg 5,6<br>Data analysis: pg 6
Describe the design in terms of the purpose, priority and sequence of methods	Study design: pg 4,5<br>Data collection: pg 5,6<br>Data analysis: pg 6
Describe each method in terms of sampling, data collection and analysis	Data source: pg 4,5<br>Sample size estimation: pg 5<br>Data collection: pg 5,6<br>Data analysis: pg 7
Describe where integration has occurred, how it has occurred and who has participated in it	Data analysis: pg 6<br>Data integration: pg 7
Describe any limitation of one method associated with the present of the other method	Study strengths and limitations: pg 14
Describe any insights gained from mixing or integrating methods	Study strengths and limitations: pg 14
[ⱡ]O'Cathain A, Murphy E, Nicholl J. The quality of mixed methods studies in health services research. J Health Serv Res Policy. 2008;13: 92-98.	
